# Denosumab Therapy for Refractory Hypercalcemia Secondary to Squamous Cell Carcinoma of Skin in Epidermolysis Bullosa

**DOI:** 10.14740/wjon907w

**Published:** 2015-04-12

**Authors:** Dinesh Giri, Renuka Ramakrishnan, James Hayden, Lynda Brook, Urmi Das, M. Zulf Mughal, Peter Selby, Poonam Dharmaraj, Senthil Senniappan

**Affiliations:** a Department of Paediatric Endocrinology, Alder Hey Children’s Hospital, Liverpool L12 2AP, UK; b Department of Paediatric Oncology, Alder Hey Children’s Hospital, Liverpool L12 2AP, UK; c Department of Paediatric Palliative Care, Alder Hey Children’s Hospital, Liverpool L12 2AP, UK; d Department of Paediatric Endocrinology, Royal Manchester Children’s Hospital, Oxford Rd, Manchester M13 9WL, UK; e Department of Medicine, Manchester Royal Infirmary, Manchester M139WL, UK; f The authors contributed equally to this manuscript.

**Keywords:** Denosumab, Hypercalcemia, Squamous cell carcinoma

## Abstract

Hypercalcemia secondary to malignancy is rare in children and the majority is caused by tumor-produced parathyroid hormone-related protein (PTHrP). We report a case of hypercalcemia refractory to bisphosphonate and corticosteroid therapy, but responsive to denosumab. A 17-year-old boy with epidermolysis bullosa (EB) and advanced squamous cell carcinoma (SCC) of the left leg was referred with severe hypercalcemia (serum calcium, 4.2 mmol/L). The serum parathyroid hormone (PTH) was 0.7 pmol/L (1.1 - 6.9 pmol/L). The hypercalcemia was initially managed with hyperhydration, prednisolone and pamidronate. Following two infusions of pamidronate (1 mg/kg/dose), serum calcium fell to 2.87 mmol/L. However the hypercalcemia relapsed within a week (serum calcium, 3.61 mmol/L) needing aggressive management with intravenous fluids, prednisolone and two further doses of pamidronate. The serum calcium fell to 2.58 mmol/L over the first 4 days, but rose to 3.39 mmol/L 3 days later. As the hypercalcemia was refractory to bisphosphonate treatment, a trial dose of subcutaneous denosumab (60 mg) was administered following which the calcium fell to 2.86 mmol/L within 24 h and normocalcemia was sustained 4 days later. We report a case of refractory hypercalcemia secondary to malignant SCC, which responded well to denosumab therapy. To our knowledge, this is the first case of hypercalcemia of malignancy in an adolescent managed with denosumab.

## Introduction

Hypercalcemia in an advanced malignant neoplasm increases the morbidity and may indicate a poor prognosis. Bone resorption and renal calcium retention are increased by parathyroid hormone-related protein (PTHrP) secreted by malignant cells. This can be treated with bisphosphonate therapy but may fail to respond or relapse [1].

We report a case of hypercalcemia in a boy with epidermolysis bullosa (EB) and malignant squamous cell carcinoma (SCC), refractory to bisphosphonate and corticosteroid therapy, but showing a promising response to denosumab therapy. EB is a heterogeneous group of congenital diseases, manifesting with blistering and erosion of skin and mucous membranes. Blister formation is usually in response to rubbing or frictional trauma. The level of tissue separation within the cutaneous basement membrane zone helps in classifying EB into three major categories: EB simplex (EBS), junctional EB (JEB) and dystrophic EB (DEB) [2].

Depending upon the type of the tumor, hypercalcemia occurs in 20-30% of adult patients with malignancy during the course of the disease depending upon the type of the tumor [3]. Hypercalcemia in SCC of the skin is very rare [4]. In the pediatric population hypercalcemia is very rare and occurs only in 0.4-0.7% of childhood malignancies [5]. However, this can have a major impact on the quality of life for these children, often necessitating inpatient stay for hydration and bisphosphonate therapy.

Intravenous fluids and bisphosphonates are the main modalities of management of hypercalcemia of malignancy and high success rates have been reported [6]. Denosumab is known to be a potent inhibitor of osteoclast development, activation and survival and has been used in the management of a few adult patients with hypercalcemia secondary to malignancy [7]. Denosumab has been used to treat two children with post-transplantation hypercalcemia in osteopetrosis [8]. Denosumab treatment has also been reported in a 9-year-old boy with severe fibrous dysplasia [9]. However, this has never been used in children with hypercalcemia of malignancy.

## Case Report

A 17-year-old boy with an advanced SCC of the left leg and EB was referred to the endocrine department, with hypercalcemia. The diagnosis of EB was made during infancy. The SCC had previously been surgically excised but recurred 3 years later, with extensive multiple leg lesions which were not amenable to surgery and definitive treatment due to the advanced stage of the neoplasm.

Cetuximab (recombinant, human/mouse chimeric monoclonal antibody (MAb)) which specifically binds to the extracellular domain of the human epidermal growth factor receptor (EGFR) was initiated as a part of this patient’s management with the aim of improving his symptoms, but unfortunately showed no evidence of efficacy with respect to disease or symptom control [10]. It has been shown to result in disease control in 69% of patients with SCC in a phase 2 study [11]. His subsequent management focused on palliative needs as directed by the patient, aiming to control symptoms thereby enabling quality of life. A bone scan to look for bony metastases was not performed, as the malignancy was advanced and it was felt that it was unlikely to add to the medical management.

### Week 1

The peak serum calcium concentration at the time of referral was 4.2 mmol/L (normal range: 2.25 - 2.74 mmol/L). The patient was symptomatic with limb pain and severe vomiting possibly related to hypercalcemia. The plasma urea was 3.3 mmol/L (2.5 - 6.7 mmol/L) and creatinine was 43 µmol/L (64 - 108 µmol/L). The patient’s previous bone profile was within the normal limits. Parathyroid hormone (PTH) was appropriately suppressed at 0.7 pmol/L (1.1 - 6.9 pmol/L). The other investigations include phosphate 0.83 mmol/L (0.74 - 1.15 mmol/L), and alkaline phosphatase 2,500 IU/L (203 - 1,151 IU/L). The serum 25 hydroxy vitamin D level was slightly low at 31 nmol/L. Serum PTHrP was elevated at 2.1 pmol/L (0.0 - 1.8 pmol/L), and 1,25-dihydroxyvitamin D concentration was raised at 173 pmol/L (43 - 143 pmol/L) (Table 1).

**Table 1:** Serum Concentrations of Various Parameters at Presentation

Corrected calcium (mmol/L)	4.2 (2.25 - 2.74)
Phosphate (mmol/L)	0.83 (0.74 - 1.53)
Alkaline phosphatase (IU/L)	2,500 (203 - 1,151)
Parathyroid hormone (pmol/L)	0.7 (1.1 - 6.9)
25 Hydroxy vitamin D (nmol/L)	31 (> 50)
PTHrP (pmol/L)	2.1 (0.0 - 1.8)
1,25 Hydroxy vitamin D (pmol/L)	173 (43 - 143)

**Figure 1:**
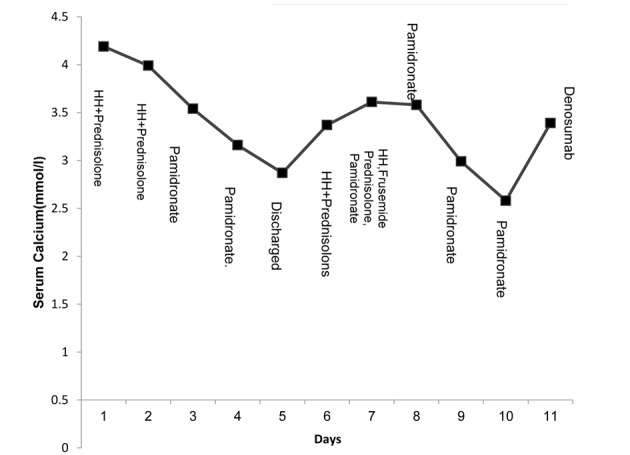
Initial management of hypercalcemia (week 1 and week 2). HH denotes hyperhydration.

The initial management of hypercalcemia consisted of hyperhydration with twice maintenance intravenous fluids (0.9% normal saline), and a dose of prednisolone (1 mg/kg). There was a marginal improvement of serum calcium (4.19 mmol/L to 3.99 mmol/L) before a second dose of prednisolone was given. Over the next 48 h, two doses of pamidronate were administered, one at 0.5 mg/kg and one at 1 mg/kg. Serum Calcium fell to 3.54 mmol/L and then stabilized at 2.87 mmol/L by 7 days after infusion. At this point, the hypercalcemia was thought to have responded to pamidronate and the patient was discharged home with further plans to monitor serum calcium in a week (Fig. 1).

### Week 2

The boy was readmitted with a febrile illness and cough secondary to lower respiratory tract infection, requiring antibiotic treatment. At this point the serum calcium was 3.61 mmol/L. He was once again managed with intravenous fluids, corticosteroids and intravenous pamidronate (1 mg/kg). The hyperhydration caused excess fluid retention, for which intravenous furosemide (1 mg/kg) was administered to facilitate fluid loss by diuresis and also with the aim of promoting calcium excretion in the urine. The patient was maintained on regular prednisolone (1 mg/kg/day).

**Figure 2:**
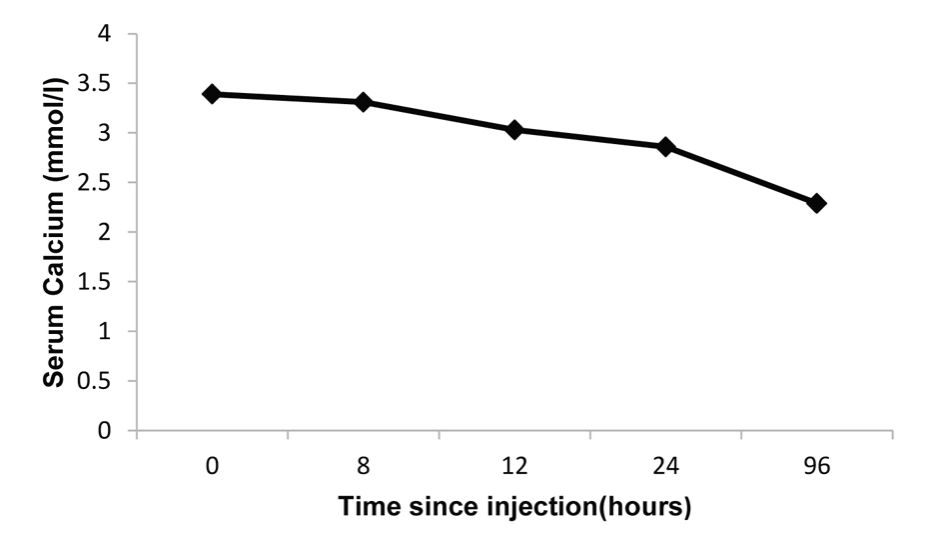
Response to denosumab (60 mg subcutaneous injection).

Following the above intensive management, the plasma calcium fell to 2.58 mmol/L over the first 4 days, but rose to 3.39 mmol/L 3 days later (Fig. 1). As the patient had failed to respond to conventional treatment strategies, a trial dose of denosumab at a dose of 60 mg was given subcutaneously. The serum calcium concentration started to fall gradually 8 h post-denosumab and 24 h later the serum calcium concentration was 2.87 mmol/L. Four days later, there was a sustained fall in serum calcium to 2.29 mmol/L which later continued to stay within the normal range (Fig. 2).

## Discussion

SCC is an epithelial malignancy and can involve multiple anatomical sites. PTHrP-related hypercalcemia or humoral hypercalcemia of malignancy has been reported in SCC of the lung [12]. PTHrP is secreted by the malignant cells. The PTH receptors in bone and kidney are targeted in a similar way by PTHrP, which in turn stimulates osteoclastic bone resorption, increases renal calcium reabsorption and promotes the formation of 1,25-dihydroxyvitamin D [13]. The combined effect of PTHrP in promoting bone resorption, mobilization and reducing renal excretion of calcium can cause a rapid increase in serum calcium. Conventional treatment methods with hyperhydration and bisphosphonates may not be effective in such situations. The PTHrP concentrations are not usually affected and the stimulus for enhanced bone resorption is maintained which can result in bisphosphonates not having a long lasting effect on bone resorption [14]. Calcimimetics and bisphosphonates have been used in controlling hypercalcemia related to SCC of the lung [14]. Successful treatment of refractory hypercalcemia with high dose denosumab has been reported in an adult with recurrent parathyroid carcinoma [15].

At present, bisphosphonates represent the drug of choice for treating patients with hypercalcemia of malignancy [16]. They inhibit osteoclasts, induce apoptosis in these cells and bind to bone, blocking osteoclastic resorption and osteolysis [17]. Corticosteroids exert their calcium decreasing action by reducing the high calcitriol production by the activated macrophages in a malignant disease.

Denosumab is a fully human monoclonal antibody and binds to and neutralizes RANKL (receptor activator of nuclear factor kappa-B ligand). Denosumab can inhibit the function of the osteoclasts which can prevent generalized bone resorption and local bone destruction [18]. Denosumab is a novel inhibitor of osteoclast function that is approved in many countries for the treatment of post-menopausal women with osteoporosis, for patients at high risk for fractures, for the prevention of skeletal related events in patients with bone metastases from solid tumors and unresectable giant cell tumors [19]. It is hypothesized that tumor cells in the bone lead to increased expression of RANKL on osteoclasts and their precursors. RANKL is an essential mediator of osteoclast function, formation, and survival [20]. Excessive RANKL-induced osteoclast activity results in resorption and local bone destruction with evidence of elevated levels of bone turnover markers [21].

Symptomatic hypocalcemia may result from denosumab in hypercalcemia of malignancy. Two randomised trials have demonstrated a higher rate of hypocalcemia with denosumab compared with zoledronic acid in patients with advanced malignancies [22]. In a single arm international study, it was found that denosumab lowered serum calcium in 64% of patients within 10 days despite recent intravenous bisphosphonate treatment [23].

Denosumab has been used successfully in adult patients with hypercalcemia of malignancy. The aim in our patient was to give symptomatic relief of hypercalcemia as part of palliative care. It aided him going home. He was comfortable and was able to participate in some activities. It added, indeed enabled, a quality of life. This was achieved with denosumab, which resulted in steady fall of plasma calcium concentration. Measurement of plasma calcium within the first few days after starting denosumab did not show hypocalcemia, although we acknowledge the limitation that longer term surveillance was not possible due to the patient’s demise. Denosumab could also be used in patients with hypercalcemia where bisphosphonates are contraindicated due to impaired renal function [24].

### Conclusion

We report our experience in managing a young patient with refractory hypercalcemia secondary to SCC of skin using denosumab. In our patient, the sustained fall of calcium was noted following denosumab therapy. Denosumab could be considered as a treatment option in children with refractory hypercalcemia.
